# Unraveling rare form of adult-onset NIID by characteristic brain MRI features: A single-center retrospective review

**DOI:** 10.3389/fneur.2022.1085283

**Published:** 2022-12-15

**Authors:** Fan Li, Qi Wang, Ying Zhu, Jiangxi Xiao, Muliang Gu, Jiaxi Yu, Jianwen Deng, Wei Sun, Zhaoxia Wang

**Affiliations:** ^1^Department of Neurology, Peking University First Hospital, Beijing, China; ^2^Medical Imaging Department, Peking University First Hospital, Beijing, China; ^3^Beijing Key Laboratory of Neurovascular Disease Discovery, Beijing, China

**Keywords:** neuronal intranuclear inclusion disease (NIID), MRI, ribbon sign, leukoencephalopathy, myopathy

## Abstract

Adult-onset neuronal intranuclear inclusion disease (NIID) is a rare neurodegenerative disorder with high clinical heterogeneity. Previous studies indicated that the high-intensity signals in the corticomedullary junction on diffusion-weighted imaging (DWI) on brain MRI, known as the “ribbon sign,” could serve as a strong diagnostic clue. Here we used the explorative approach to study the undiagnosed rate of adult-onset NIID in a single center in China *via* searching for the ribbon sign in picture archive and communication system (PACS) and report the clinical and radiological features of initially undiagnosed NIID patients.

Consecutive brain MRI of 21,563 adult individuals (≥18 years) in the PACS database in 2019 from a tertiary hospital were reviewed. Of them, 4,130 were screened out using the keywords “leukoencephalopathy” and “white matter demyelination.” Next, all 4,130 images were read by four neurologists. The images with the suspected ribbon sign were reanalyzed by two neuroradiologists. Those with the ribbon sign but without previously diagnosed NIID were invited for skin biopsy and/or genetic testing for diagnostic confirmation. The clinical features of all NIID patients were retrospectively reviewed.

Five patients with high-intensity in the corticomedullary junction on DWI were enrolled. Three patients were previously diagnosed with NIID confirmed by genetic or pathological findings and presented with episodic encephalopathy or cognitive impairment. The other two patients were initially diagnosed with limb-girdle muscular dystrophy (LGMD) with rimmed vacuoles (RVs) and normal pressure hydrocephalus (NPH) in one each. Genetic analysis demonstrated GGC repeat expansion in the NOTCH2NLC gene of both, and skin biopsy of the first patient showed the presence of intranuclear hyaline inclusion bodies. Thus, five of the 21,563 adult patients (≥18 years) were diagnosed with NIID. The distinctive subcortical high-intensity signal on DWI was distributed extensively throughout the lobes, corpus callosum, basal ganglia, and brainstem. In addition, T2-weighted imaging revealed white matter hyperintensity of Fazekas grade 2 or 3, atrophy, and ventricular dilation. Distinctive DWI hyperintensity in the junction between the gray and white matter can help identify atypical NIID cases. Our findings highly suggest that neurologists and radiologists should recognize the characteristic neuroimaging pattern of NIID.

## 1. Introduction

Adult-onset neuronal intranuclear inclusion disease (NIID) is a rare neurodegenerative disease with distinctive histological and MRI features. Its clinical features show great heterogeneity, and it can be classified into infantile-, juvenile-, and adult-onset subgroups according to age at onset (1–5). Adult-onset NIID often manifests as episodic encephalopathy, cognitive impairment, limb weakness, and autonomic dysfunction (6). Due to the availability of skin biopsy and the recent identification of genetic causes, the diagnostic efficiency of the disease has improved greatly (7). The pathological feature of NIID is the appearance of eosinophilic intranuclear inclusions in cells of various systems, including neurons, visceral organ cells, and the skin (8). The causative mutation of NIID was the expansion of the CGG repeat sequence in 5′ untranslated region of the *NOTCH2NLC* gene (1).

Increasing studies have reported that NIID shows a wide phenotypic spectrum. In particular, the diagnosis is challenging in cases with atypical manifestations (7, 9). Thus, recognizing characteristic clinical signs or symptoms is very helpful in the diagnostic workup of adult-onset NIID, as it can help guide specific laboratory tests. In 2014, Sone et al. reported that a high intensity of the corticomedullary junction on diffusion-weighted imaging (DWI) (10). Forming the “ribbon sign” or “diaper sign” (11, 12), is a highly characteristic finding of NIID. Since then, multiple studies have shown similarly distinctive imaging features of NIID (6, 8, 11, 13–24). Diffuse high intensity in the cerebral white matter on FLAIR is known to be other characteristic radiological features of NIID (8, 11). A study from Singapore also revealed that previously undiagnosed patients with NIID can be identified by searching for abnormalities on DWI in picture archive and communication system (PACS) (20). Here we aimed to retrospectively analyze brain MRI in a single center in China to identify under- or misdiagnosed NIID cases and describe the clinical features of these atypical cases.

## 2. Methods

This was an explorative study approved by the Human Research Ethics Committee of Peking University First Hospital. All patients provided informed consent for this study.

### 2.1. PACS report review and patient enrollment

Four neurologists (one with 3 years, one with 10 years, and two with more than 20 years of specialist experience, respectively) who were blinded to clinical information retrospectively identified patients (≥18 years old) with subcortical white matter abnormalities in the PACS radiology report database from 1 January 2019 to 30 December 2019 of Peking University First Hospital, a tertiary Class A hospital in China. The neurologists had received training of NIID images reading and PACS operation before performing the evaluations for 3 years. A list of candidate patients was generated from brain MRI reports containing the keywords “leukoencephalopathy” and “white matter demyelination.” Those with the ribbon sign on DWI with or without leukoencephalopathy were enrolled, while those with white matter demyelination but without DWI hyperintensity were excluded. The images of all enrolled patients were reanalyzed by two professional neuroradiologists (with more than 20 years of specialist experience).

**Figure 1:**
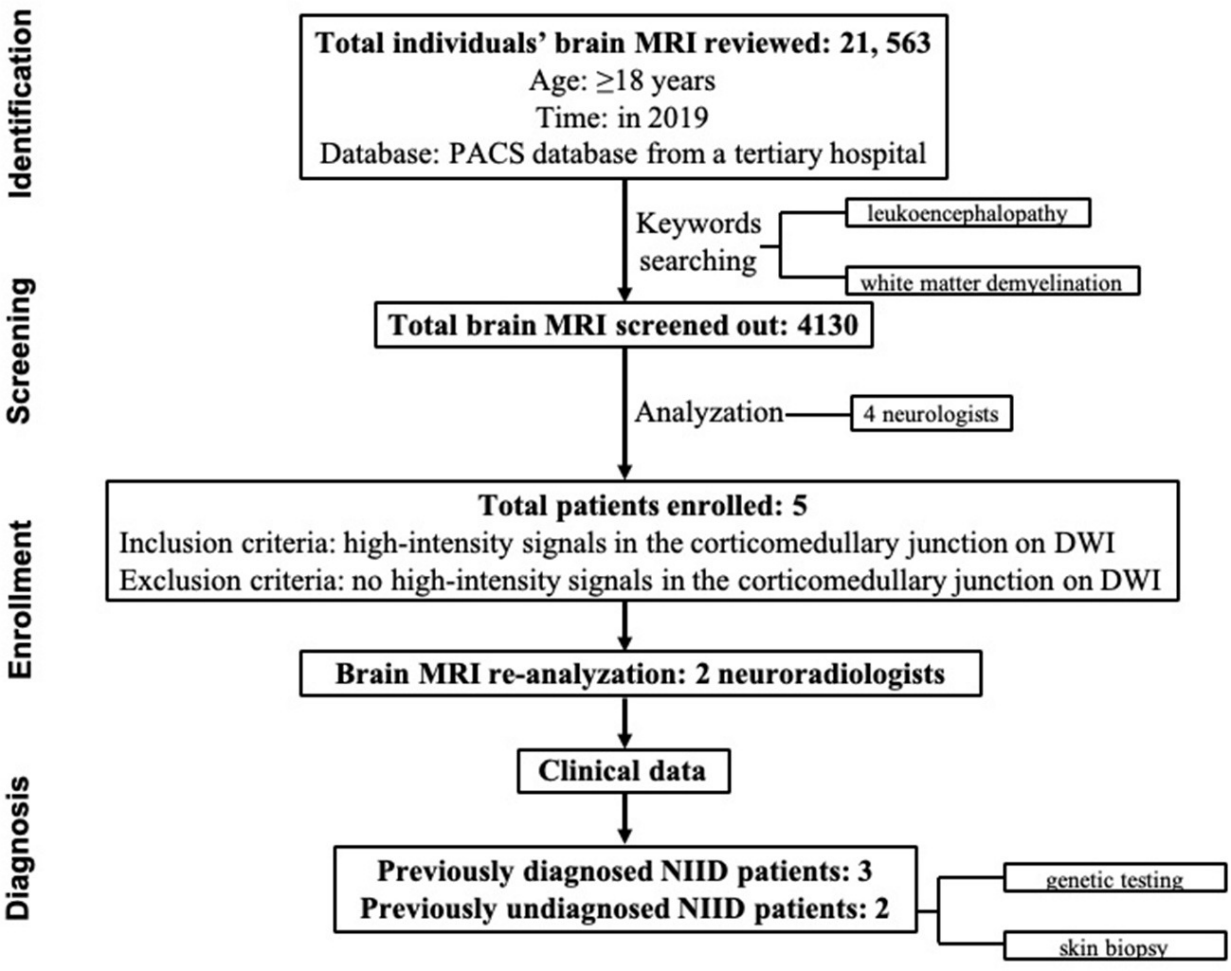
Flowchart of the study enrollment procedure.

From the list of enrolled patients, the blind was uncovered and the clinical data were reviewed, information was collected regarding age at onset, disease progression, other clinical manifestations, family history, and a series of laboratory tests. All of the patients with a suspected diagnosis of NIID were followed by neurologists and underwent skin biopsy and/or genetic analysis (Figure 1).

### 2.2. Brain MRI

All MRI examinations were performed as routine clinical care with 3.0 T or 1.5 T MRI scanner. Conventional T1-weighted spin-echo (T1WI) sequences, T2-weighted spin-echo (T2WI) sequences, or fluid-attenuated inversion recovery (FLAIR) sequences and gradient-recalled echo, DWI, and apparent diffusion coefficient were performed according to routine procedures. All images were 5-mm thick with 1-mm spacing. Each patient's Fazekas grade was categorized into 3 severity groups: grade 1 (mild changes), single lesions <10 mm and/or “grouped” lesions <20 mm in any diameter; grade 2 (moderate changes), single hyperintense lesions 10–20 mm and hyperintense areas linked by no more than “connecting bridges” >20 mm in any diameter; and grade 3 (severe changes), both single and confluent hyperintense areas of ≥20 mm in any diameter (25).

### 2.3. Pathological examinations of skin biopsy

Skin biopsy was performed in the distal part of the leg (10 cm above the external malleolus) of previously undiagnosed patients with their informed consent. Part of the specimen was fixed using 4% formalin solution, embedded in paraffin, cut into 4-mm-thick sections, and stained with hematoxylin and eosin. The immunohistochemical and immunofluorescent stains were performed with anti-p62 antibody (sc-28359; Santa Cruz Biotechnology). Some of the specimens was fixed in 2.5% glutaraldehyde and 1% osmium tetroxide, and then embedded in Epon812. Ultrathin sections were examined under electron microscopy. To increase the positive rate of intranuclear inclusions, ultrastructural examinations were performed three times.

### 2.4. Genetic screening

Repeat-primed polymerase chain reaction (RP-PCR) was performed as described in our previous study to identify the repeat expansion in the *NOTCH2NLC* gene in undiagnosed patients (3). The PCR primer mix contained NOTCH2NLC-F: 5′-FAM-GGCATTTGCGCCTGTGCTTCGGACCGT-3′; M13-(GGC)4(GGA)2-R: 5′-CAGGAAACAGCTATGACCTCCTCCGCCGCCGCCGCC-3′; and M13-linker-R: 5′-CAGGAAACAGCTATGACC-3′. A saw-tooth tail pattern in the electropherogram was considered the disease-associated repeat expansion. Fluorescence amplicon length PCR (AL-PCR) was used to detect the length of the GGC repeat expansion. The composition of PCR mix was identical to that of RP-PCR except for the use of 50 ng of genomic DNA as a template and a different primer pair of NOTCH2NLC-AL-F: 5′-VIC-CATTTGCGCCTGTGCTTCGGAC-3′; and NOTCH2NLC-AL-R: 5′-AGAGCGGCGCAGGGCGGGCATCTT-3′. The PCR conditions were the same as those for RP-PCR. Electrophoresis was performed on a 3,500 × l Genetic Analyzer (Thermo Fisher Scientific, Waltham, MA, USA) and the data were analyzed using GeneMapper software (Thermo Fisher Scientific). The length of the highest signal peak of the expanded allele was used to calculate the repeat number.

## 3. Results

### 3.1. PACS report search and case selection

A total of 4,130 (19.15%) cases were identified using the “leukoencephalopathy” or “white matter demyelination” keywords among 21,563 patients ≥18 years of age who underwent brain MRI between 1 January 2019 and 30 December 2019. Among the 4,130 patients, five (0.12%) had high-intensity areas in the corticomedullary junction on DWI. Review of their clinical records revealed that three patients (Patients 1–3) had previously confirmed NIID (Table 1). The remaining two patients were diagnosed with rimmed vacuolar myopathy (Patient 4) and normal pressure hydrocephalus (Patient 5). The clinical and neuroimaging data of the five patients are summarized in Table 1.

**Table 1:** Clinical and MRI findings in 5 patients with NIID identified from PACS.

**33.8-1.55,15.5Patient** **number/sex/age** **(year)**	**Initial clinical assessment**	**Final diagnosis**	**Onset** **symptoms**	**Family history**	**Location of DWI** **hyperintensity**	**Fazekas grade**	**Hyperintensity** **on T2/FLAIR** **images**
1/M/54	NIID	NIID	Episodic encephalopathy	+	F, P, T, O	3	F, P, T, O BG, CC BS, MCP, C
2/F/63	NIID	NIID	Cognitive impairment	–	F	2	F, P, T, O CC
3/F/60	NIID	NIID	Episodic encephalopathy	–	F, P, T	2	F, P, T, O CC
4/F/63	Myopathy	NIID	Muscular weakness	–	F, P, T, O BG, CC BS, MCP	3	F, P, T, O BG, CC BS, MCP, C
5/F/78	NPH	NIID	Urinary incontinence	+	F, P, T, O BG, CC BS	3	F, P, T, O BG, CC BS, C

F, frontal; P, parietal; T, temporal; O, occipital; BG, basal ganglion; CC, corpus callosum; BS, brainstem; MCP, middle cerebellar peduncle; C, cerebellum.

### 3.2. Genetic results

Patients 4 and 5 were diagnosed with NIID through genetic analysis during follow-up, and the result showed GGC repeat expansion of *NOTCH2NLC*.

### 3.3. Skin biopsy

**Figure 2:**
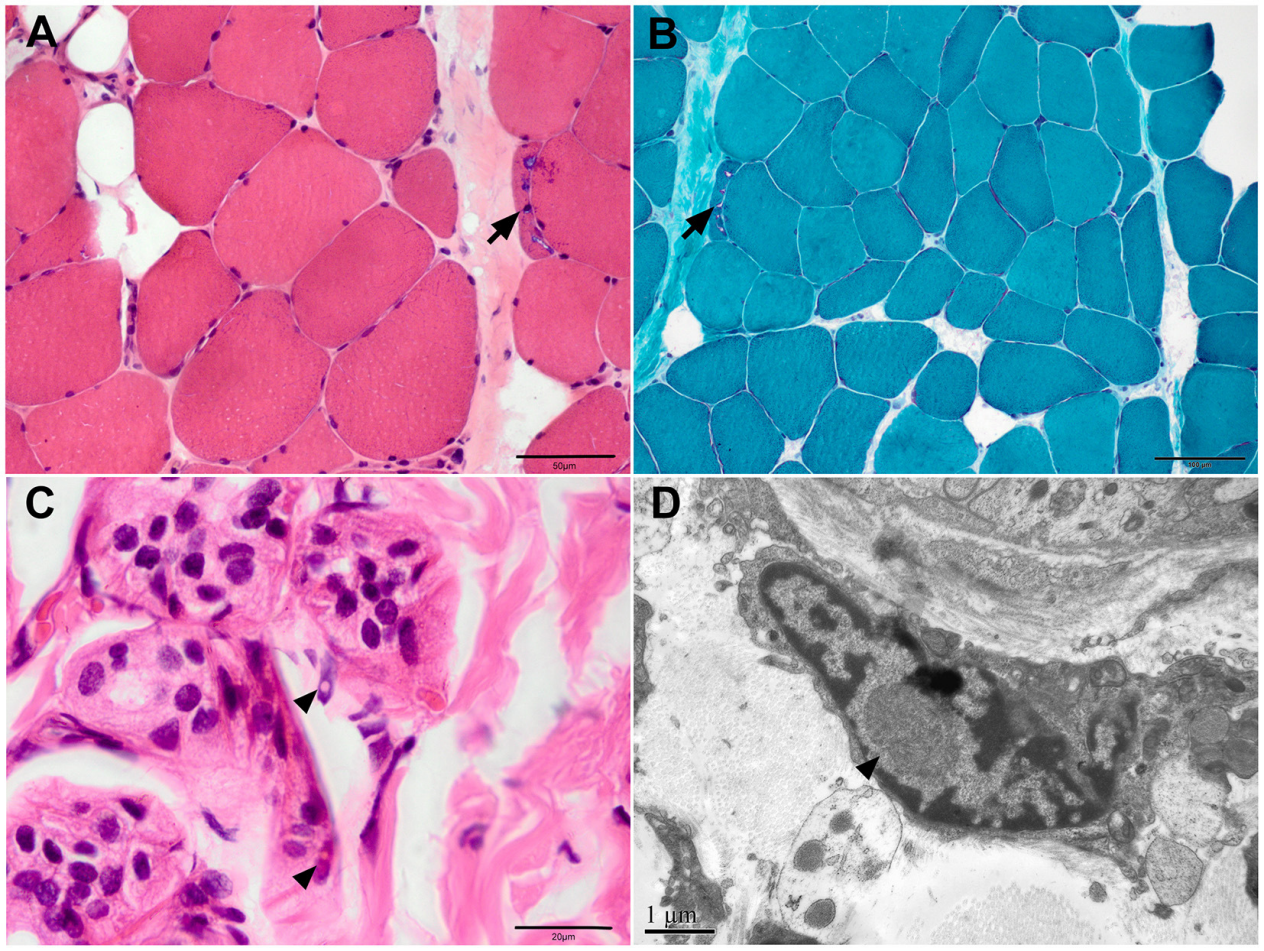
Muscle and skin pathology of Patient 4. **(A)** Haematoxylin and eosin (HE), **(B)** modified Gomori trichrome staining on muscle samples showed myopathic changes with rimmed vacuoles (arrows). **(C)** HE staining and **(D)** electron microscopy of skin sections showed inclusions in the fibroblasts (arrowhead). Scale bars = 1μm **(D)**, 20μm **(C)**, 50μm **(A)**, 100μm **(B)**.

Patient 4 consented to skin biopsy. The pathological result showed characteristic inclusions with P62 and ubiquitin antibody positivity in the nuclei of sweat gland cells (Figures 2C, D).

### 3.4. Clinical data of atypical NIID patients

#### 3.4.1. Patient 4

A 63-year-old woman with a 20-year history of progressive symmetrical muscle weakness was admitted to our department. She complained of difficulty squatting and climbing stairs accompanied by myalgia at 43 years of age and weakness of the arms at 46 years of age. Her medical history was unremarkable except for a 10-year history of hypertension. On admission, the physical examination revealed symmetrical muscle weakness of the proximal limbs and neck muscles and postural tremor of the head and hands. Mental status examination evaluated normal cerebral cognitive function. The serum creatine kinase level was mildly elevated. Electrophysiological study revealed the myopathic pattern in the muscles with decreased nerve conduction velocity of the bilateral median nerves. Mini-Mental State Examination and Montreal Cognitive Assessment at 60 years of age revealed no cognitive impairment. Findings on muscle MRI were characteristic of fatty infiltration and atrophy of the hip and thigh muscles. Muscle biopsy showed both myopathic changes including rimmed and non-rimmed vacuoles in some fibers and neurogenic changes with fiber-type grouping and group atrophy. Oil Red O staining showed only a mild increase of lipid droplets (Figures 2A, B). Next-generation sequencing revealed no pathogenic variants in the myopathy-related genes. The patient had been misdiagnosed with limb-girdle muscular dystrophy or lipid storage myopathy.

**Figure 3:**
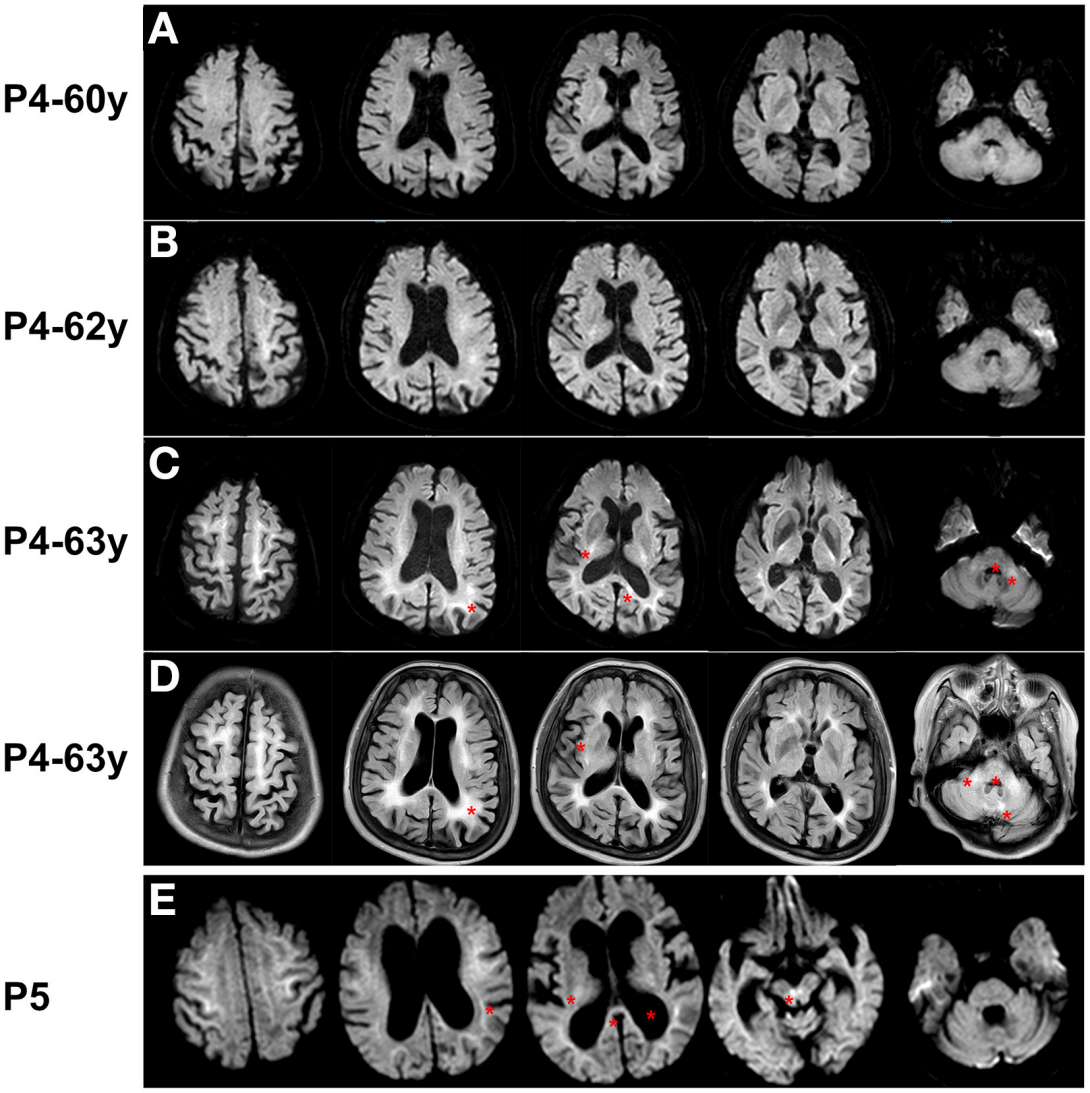
Brain MRI of Patient 4 and Patient 5. **(A–C)** DWI sequence of Patient 4 at age 60, 62, and 63 years showed irreversible hyperintensity in the corticomedullary junction in frontal, parietal, temporal, and occipital lobes, peri-globus pallidus, left splenium of the corpus callosum, deep lobar white matter of the corona radiata, pons, and branchium pontis. **(D)** FLAIR sequence of Patient 4 at age 63 years revealed hyperintensity corresponding with Fazekas grade 3 staging in subcortical regions, corpus callosum, external capsule, pons, branchium pontis, and paravermal areas of cerebellum. **(E)** DWI sequence of Patient 5 showed ribbon sign in frontal, parietal, temporal, and occipital lobes, and high-intensity signals in thalamus, splenium of the corpus callosum, and the, and dilated ventricles (asterisks).

Brain MRI performed at age 60, 62, and 63 years revealed progressive DWI lesions, leukoencephalopathy, and cerebral atrophy (Figures 3A–D). DWI sequencing showed irreversible hyperintensity in the subcortical region at the junction between the gray matter and the white matter of the bilateral frontal, parietal, temporal, and occipital lobes in succession. The basal ganglia (peri-globus pallidus), left splenium of the corpus callosum, deep lobar white matter of the corona radiata, bilateral cerebral peduncle, pons, and branchium pontis were also affected. Notably, the signal intensity was enhanced in all regions over time. T2/FLAIR revealed hyperintensity continuously involving the entire white matter from the periventricular to the deep or subcortical U-fibers of the frontal, parietal, temporal, and occipital lobes corresponding with Fazekas grade 3 staging. High signal intensity on T2/FLAIR was also observed in the corpus callosum, external capsule, pons, branchium pontis, and paravermal areas of cerebellum. Other abnormalities included generalized cerebral and cerebellar atrophy. MR angiography showed no focal occlusion or stenosis.

#### 3.4.2. Patient 5

A 76-year-old woman was referred to neurosurgery for a 19-year history of urinary incontinence and a 5-year history of cognitive impairment and walking instability. She complained of slow walking, a lower foot height, and poor balance. Furthermore, she had memory loss and was slow to respond. In recent years, she reported feeling sleepy or confused with an intermittent fever from which she recovered in 1–2 days. Her symptoms had gradually worsened, and she had been dependent on family members to help her walk for the past year. Her medical history included diabetes, cataracts, and retinitis pigmentosa. A sister had a 30-year history of urinary incontinence. Brain computed tomography and MRI showed ventriculomegaly, for which she was admitted with the preliminary diagnosis of normal pressure hydrocephalus (NPH). However, the cerebrospinal fluid tap-test was negative, so the diagnosis of probable NPH was not supported.

Reanalysis of the DWI sequence showed high-intensity signals in the corticomedullary junction of the bilateral frontal, parietal, temporal, and occipital lobes, and the thalamus, splenium of the corpus callosum, and the ventral tegmental area (Figure 3E). On T2/FLAIR, the global Fazekas grading was grade 3. The hyperintensity continuously involved the entire white matter from the periventricular to the deep or subcortical U-fibers of the diffuse lobes. The thalamus, corpus callosum, and pons were also involved. Other signs included cerebral ventricular dilation, especially of the lateral and third ventricles.

## 4. Discussion

In recent years, the number of patients diagnosed with NIID has substantially increased with the application of skin biopsy and *NOTCH2NLC* genetic testing (3, 5, 26). Meanwhile, the recognition of characteristic changes also plays an indisputable role in the improved diagnostic yield (27). A symmetrical high-intensity signal in the corticomedullary junction on DWI and a remarkable leukoencephalopathy observed in the MRI strongly support a NIID diagnosis (11). A Singapore study emphasized that previously undiagnosed patients with NIID can be identified by abnormalities in the junction between the gray and white matter on DWI in PACS and subsequently confirmed by skin biopsy; the minimum missed diagnostic rate is 25.00% (20). In this study, we identified five patients with characteristic MRI features of NIID among 4,130 cases of leukoencephalopathy or white matter demyelination in a tertiary hospital PACS in China, of which three were previously diagnosed NIID patients and two were missed NIID patients confirmed by following skin biopsy and /or genetic testing. Thus, the missed diagnosis rates of NIID in adults with white matter high-intensity signaling (4,130 cases) and the distinctive ribbon sign (five cases) during routine diagnostic workups in our center were 0.05 and 40%, respectively.

Among the three previously diagnosed NIID patients, two initially presented with episodic encephalopathy and one with cognitive impairment. Previous study revealed that the onset symptoms in adult-onset NIID included episodic encephalopathy, bladder dysfunction, cerebellar ataxia, tremor, parkinsonism, migraine, and visual abnormalities, while progressive cognitive impairment could be the main classical symptom in patients first diagnosed at around 60 years of age (5, 11).

It is noteworthy that two patients without a previous diagnosis were identified by this MRI re-evaluation screening process. Patient 4 was misdiagnosed for years because of the performance as a rare myopathy phenotype without cognitive and autonomic impairment. Sone et al. demonstrated that the limb weakness–dominant NIID group consisted of familial cases with an age at onset of <40 years and with larger-size repeat expansions. Dementia and leukoencephalopathy were relatively mild and did not present before a 20-year disease duration (11). They usually began in the distal part of lower limbs, moved up to the throat muscles and face, and can also affect the cardiac muscle (5, 28). Other reports about NIID-related muscular manifestations included ophthalmoplegia and myasthenia (29). We demonstrated that myopathy could be the only presenting symptom in sporadic adult-onset NIID cases. This interesting phenomenon suggested that brain MRI may be useful in some unexplained cases of myopathy in which muscle biopsy showed vacuoles accompanied by neurogenic alterations.

A longstanding urinary disturbance may be the first neurological symptom of NIID patients (30). In Patient 5, the urinary incontinence preceded the abnormal gait and cognitive decline, and combined with the brain atrophy on the imaging, NPH was clinically diagnosed. However, it is worth noting that the episodic encephalopathy suggests the probable symptom of NIID rather than NPH. It should also be addressed that the clinical manifestations of a few NIID patients are quite atypical and easily misdiagnosed. The recognition of characteristic MRI features could improve the detection rate of NIID.

On conventional DWI sequences, the corticomedullary junction of the frontal, parietal, and temporal lobes was affected in more patients than the occipital lobe (16). We found extensive lobar distribution, which was not consistent with sparing of the occipital lobe and perirolandic region like in the Singapore cohort (20). The neuroimaging features of NIID involve dynamic changes in patients with encephalitic episodes (31). The longitudinal disease progression noted in Patient 4 showed progressive involvement of the frontal and parietal lobes, followed by the temporal lobe, and finally the occipital lobes on DWI of NIID. We speculated that NIID without episodic symptoms showed progressive DWI hyperintensity and that repeat brain MRI could assist the diagnosis. Hyperintensity DWI signals were not limited to the corticomedullary region. We also reported involvement of the corpus callosum (particularly the splenium) and middle cerebellar peduncles on DWI consistent with previous studies (11, 20). Unlike other investigators, we found DWI abnormalities of the peri-globus pallidus and the thalamus in the basal ganglia.

Uniform and symmetrical leukoencephalopathy, especially those in the centrum semiovale and corona radiata, are predominant MRI features of adult-onset NIID. Using the genetic analysis, a previous Japanese study referred the patients with GGC expansion in *NOTCH2NLC* as the most frequent cause of adult leukoencephalopathy without acquired etiology (32). In our study, all patients had Fazekas grade 2 or 3 confluent white matter hyperintensity throughout the entire lobes and the corpus callosum, internal and external capsule, thalamus, basal ganglia, and middle cerebellar peduncles consistent with previous studies (11, 20, 33). Isolated paravermal hyperintensities on T2WI/FLAIR were reported to be the initial radiologic findings in NIID patients (34). We also found additional involvement on T2WI/FLAIR of the pons that was not previously well-reported, suggesting that extensive white matter lesions in the cerebral hemispheres brainstem and cerebellum can be involved in NIID. Pathogenically, the typical high signals arround U-fibers on DWI and leukoencephalopathy on T2WI/FLAIR are associated with the pathological spongiotic changes and diffuse myelin pallor (14). Radiologically, the mechanism of DWI abnormalities has not yet been established as being caused by cytotoxic edema, myelin edema, T2 shine through, or spongiform degeneration as was reported in a single case. In addition to mild or severe DWI abnormalities and leukoencephalopathy, brain atrophy was also observed in some patients (33). The extensive brain atrophy we identified might have been due to subsequent neuronal loss and gliosis (14, 17).

Despite ribbon sign as a sensitive diagnostic indicator, the characteristic DWI feature appears in only 37% of all NIID patients (35). Few NIID patients manifested with mild leukoaraiosis, progressive leukoencephalopathy, brain atrophy, or focal reversible leukoencephalopathy but without high-intensity signaling in the corticomedullary junction on DWI as described in previous studies (12, 31, 36–38). So, except patients with certain characteristics on DWI, patients without the ribbon sign should also be followed to avoid missed diagnosis.

However, keyword searching method of radiological reports in our study may miss out potential NIID patients whose reports did not contain the exact words. Future advanced methodologies based on images or fuzzy search could benefit. Additionally, considering small sample size and short searching duration in this study, more centers and samples are needed to reflect the missed diagnosed rates.

In conclusion, the clinical manifestations of adult-onset NIID showed significant heterogeneity. Distinctive DWI hyperintensity at the junction between the gray and white matter can improve the diagnostic rate of atypical cases. Radiologists should recognize the characteristic neuroimaging changes of this highly heterogeneous disease.

## Data availability statement

The raw data supporting the conclusions of this article will be made available by the authors, without undue reservation.

## Ethics statement

The studies involving human participants were reviewed and approved by Human Research Ethics Committee of Peking University First Hospital. The patients/participants provided their written informed consent to participate in this study.

## Author contributions

ZW and WS take full responsibility for the paper. Images screening and initial analysis were performed by FL, MG, WS, and ZW. Enrolled images were re-analyzed by YZ and JX. Material preparation, data collection, and analysis were performed by QW, JY, and JD. The first draft of the manuscript was written by QW. All authors commented on previous versions of the manuscript and contributed to the study conception and design. All authors read and approved the final manuscript.
